# Physical activity monitoring to assess disability progression in multiple sclerosis

**DOI:** 10.1177/2055217320975185

**Published:** 2020-12-07

**Authors:** Charlotte M Stuart, Aravinthan Varatharaj, Janine Domjan, Sheaba Philip, Ian Galea, SIMS study group

**Affiliations:** Clinical Neurosciences, Clinical and Experimental Sciences, Faculty of Medicine, University of Southampton, Southampton, UK; Clinical Neurosciences, Clinical and Experimental Sciences, Faculty of Medicine, University of Southampton, Southampton, UK; Wessex Neurological Centre, University Hospital Southampton NHS Foundation Trust, Southampton, UK; Wessex Neurological Centre, University Hospital Southampton NHS Foundation Trust, Southampton, UK; Clinical Neurosciences, Clinical and Experimental Sciences, Faculty of Medicine, University of Southampton, Southampton, UK; Clinical Neurosciences, Clinical and Experimental Sciences, Faculty of Medicine, University of Southampton, Southampton, UK; Wessex Neurological Centre, University Hospital Southampton NHS Foundation Trust, Southampton, UK

**Keywords:** Multiple sclerosis, progressive multiple sclerosis, remote physical activity monitoring, accelerometer, wearable electronic devices, teleneurology

## Abstract

**Background:**

Clinical outcome measurement in multiple sclerosis (MS) usually requires a physical visit. Remote activity monitoring (RAM) using wearable technology provides a rational alternative, especially desirable when distance is involved or in a pandemic setting.

**Objective:**

To validate RAM in progressive MS using (1) traditional psychometric methods (2) brain atrophy.

**Methods:**

56 people with progressive MS participated in a longitudinal study over 2.5 years. An arm-worn RAM device measured activity over six days, every six months, and incorporated triaxial accelerometry and transcutaneous physiological variable measurement. Five RAM variables were assessed: physical activity duration, step count, active energy expenditure, metabolic equivalents and a composite RAM score incorporating all four variables. Other assessments every six months included EDSS, MSFC, MSIS-29, Chalder Fatigue Scale and Beck’s Depression Inventory. Annualized brain atrophy was measured using SIENA.

**Results:**

RAM was tolerated well by people with MS; the device was worn 99.4% of the time. RAM had good convergent and divergent validity and was responsive, especially with respect to step count. Measurement of physical activity over one day was as responsive as six days. The composite RAM score positively correlated with brain volume loss.

**Conclusion:**

Remote activity monitoring is a valid and acceptable outcome measure in MS.

## Introduction

Monitoring progression in Multiple Sclerosis (MS) is essential for clinical management and research. The most widely used measure is the Expanded Disability Status Scale (EDSS), a 10-point ordinal composite scale that categorises disability based on a number of functional systems, predominantly ambulatory function.^1^ It places a lot of emphasis on lower limb physical function, with under-representation of other important functional domains, such as upper limb function, cognition, mood and fatigue. The EDSS struggles to capture progression at the higher end of the scale and has low reproducibility at the lower end.^2^ The Multiple Sclerosis Functional Composite (MSFC) is increasingly being used as an outcome measure in MS studies; it provides an improved clinical measure of disability by combining ambulation (the timed 25-foot walk, T25FW), upper limb function (a timed 9-hole peg test, 9HPT) and cognitive function (the Paced Auditory Serial Addition Test, PASAT).^3^

Clinic-based tests such as the EDSS and MSFC provide a single snapshot of disability at one point in time and in an artificial environment. People with MS may struggle to access clinical services because of travel costs, restricted mobility, or fatigue. Recently the COVID-19 pandemic highlighted the need for remote assessments. A tele-EDSS assessment has been shown to be feasible in people with mild-to-moderate MS capable of guided self-assessment,^4^ however assessment strategies are still lacking for more disabled patients. A more convenient way to gain insight into the effects of MS on daily living is to use patient-reported outcomes such as the MS impact scale (MSIS-29),^5^ however questionnaires can suffer from subjective interpretation and recall bias. There is therefore the need for an accessible, objective outcome measure that can monitor disability within a patient’s living environment. Remote activity monitoring (RAM) using wearable accelerometer devices has been shown to be feasible and to provide clinically meaningful information in MS and other neurological diseases.^6–9^ The growing importance of serial assessments to monitor progression and the rise of telemedicine have accentuated the unmet need to validate the use of RAM in clinical settings. Hence, we set out to perform a robust validation of RAM, using an arm-worn multi-sensor device as an exemplar. We assess its measurement qualities in progressive MS, alongside three well-established clinical scales (EDSS, MSFC and MSIS-29) using traditional psychometric methods. We also validate RAM against a biological readout for progression, namely brain atrophy on magnetic resonance imaging (MRI). While T2 lesion volume increase is more informative in relapsing-remitting and early progressive MS, brain atrophy is correlated with disability worsening across the entire MS course.^10,11^

## Patients and methods

### Participants

This study included adult participants (>18 years) with primary progressive (PPMS, n = 33) and secondary progressive (SPMS, n = 24) recruited to a sub-study on remote accelerometry within the Systemic Inflammation in Multiple Sclerosis (SIMS) study at the Wessex Neurological Centre, University Hospital Southampton NHS Foundation Trust. The project was covered by National Research Ethics Service Approval 12 SC 0176 and institutional research ethics approval ERGO 5562. Inclusion criteria were: (1) diagnosis of progressive MS according to the 2010 McDonald criteria^12^ (2) age ≤70 (3) EDSS ≤6.5. Exclusion criteria were: (1) relapses in the last year (2) disease-modifying or immunosuppressive treatment in the previous six months (3) comorbidities that could contribute to neurological disability.

### Clinical measures and protocol

**Figure 1.**
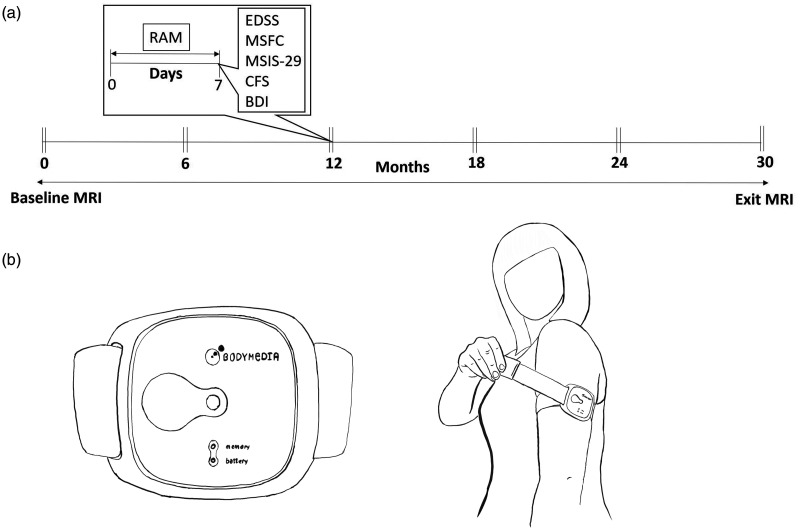
(a) Planned timeline for a study participant. At each assessment time point, participants attended two clinic visits, one week apart. The RAM device was worn between the first and second visits. The EDSS, MSFC, MSIS-29, CFS and BDI were conducted on the second visit. (b) The RAM device, an armband, was worn on the non-dominant arm.

During the first visit, participants were shown how to correctly apply the device to the back of their upper dominant arm (over the triceps) and remove it. They were instructed to wear the device for twenty-four hours a day, with the exception of swimming or bathing. Participants started wearing the RAM device at a weekday clinic visit and returned the following week. Previous studies had shown that anything between two and seven days was needed to monitor physical activity reliably with this device.^13,14^ Clinics were held on Tuesdays and Wednesdays, so the minimum wear-period was six full days, and always included the weekend. A RAM measurement week was considered valid if, on average, the device was worn for a minimum of 23 hours a day over a continuous six-day wear period, independent of number of steps per day. A week after the first visit, participants returned to the clinic for data download, height and weight measurement, completion of questionnaires (including the MSIS-29, Chalder Fatigue Scale^15^ and Beck Depression Inventory Short Form (BDI),^16^ and MSFC and EDSS assessments performed by trained and certified research staff. Follow-up duration was two and a half years, with a pair of such clinic visits every six months (six assessments in total, Figure 1(a)).

### Remote physical activity measurement

The RAM device (SenseWear Armband, Model MF-SW, Body Media, Pittsburgh, PA), shown in Figure 1(b), weighed 45 g and is worn on the arm with an adjustable strap. Two stainless steel arrays on the back house multiple sensors that measure heat flux (heat dissipated by the body), galvanic skin response (the conductivity of the skin, varying according to physical and emotional stimuli) and skin temperature. A tri-axial accelerometer measures free-living daily physical activity including upper and lower limb motion. Data from these sensors is processed by an algorithm to provide the four variables studied here, namely daily step count, metabolic equivalents (METs), physical activity duration (PhAD) and active energy expenditure (E_A_). PhAD and E_A_ were quantified for physical activity above 3.0 METs (equivalent to walking). While the Sensewear Armband has been used for physical activity outcome measurement in progressive MS patients,^17,18^ it has not been validated for detection of progression in this patient group. Sensewear Armband E_A_ measurement has been validated against indirect calorimetry in healthy populations and shown to be particularly accurate for low intensity activity.^19,20^

### Brain MRI

MRI was conducted at baseline and study exit on a 3 T MR unit (Skyra, Siemens, Erlangen, Germany) at University Hospital Southampton NHS Foundation Trust, using a 20‐element phased‐array head coil. Due to the long duration of the study, scans at entrance and exit were naturally interleaved. Structural images were acquired isotropically and used a 3 D magnetization prepared - rapid gradient echo (MP‐RAGE) sequence with the following parameters: TR = 2200 ms, TE = 2.45 ms, TI =900 ms, flip angle = 8°, GRAPPA under-sampling with parallel imaging factor = 2, field‐of‐view 250 × 250 × 176 mm^3^, voxel size 1.0 × 1.0 ×1.0 mm^3^. This sequence has excellent grey-white matter contrast, suitable for volume and atrophy estimation and tissue classification.^21^ All raw images were visually inspected by an experienced neuroradiologist; subjects with inadequate image quality (e.g. due to movement artefact) were recalled for further imaging. Two time point percentage brain volume change was estimated with SIENA,^22^ part of FSL,^23^ and then annualised. Standard-space masking was applied to improve removal of non-brain tissue,^24^ and brain extraction settings were optimised according to published recommendations.^25^ The intermediate results of brain extraction, image registration (and segmentation) were visually examined in all cases, blinded to the result of analysis, and where appropriate manual corrections applied.

### RAM variable definitions, analysis and statistics

Usability was assessed by considering patient feedback, adverse reactions, and tolerance (defined as total wear time as measured by the device, the effect of disability on wear time, and change in wear time within measurement weeks and as the study progressed). SenseWear® software (Version 7.0) was used to analyse RAM data. RAM variables were normalized to wear duration to obtain a single combined metric of physical activity. A RAM composite score was created by combining the four variables: step count, PhAD, METs and E_A_. Since the units of measurement differ across these variables, raw scores for each variable were converted to z-scores. This was after normalization of step count, E_A_ and PhAD using the cube root transformation (logarithmic transformation was insufficient for normalising the data). One study participant who was a landscape gardener with EDSS = 1.5, with very high activity and no progression on the study, was excluded from the baseline z-score calculations. The RAM score was constructed by using the simple arithmetic mean of the component variable z-scores i.e. using an equal weight for each. Correlation of individual RAM components with the overall RAM score supported its construct validity (Supplementary Table 1). MSFC component z scores were calculated relative to the mean baseline of the whole study. The physical (questions 1–20) and psychological (questions 21–29) components of the MSIS-29 are referred to here as MSIS-phys and MSIS-psych respectively.

SPSS v25 was used for statistical analyses and plots were created in GraphPad Prism v8. Results are presented for all progressive MS participants as one group, and for the separate PPMS and SPMS groups. Data distribution was determined graphically and using the Kolmogorov-Smirnov test. Responsiveness was assessed in two ways: (1) statistical significance of the change over time, using the Wilcoxon matched pair signed rank tests, comparing paired measurements separated in time (2) the effect size of the change was calculated as the Wilcoxon Z divided by the square root of the number of observations (Z/√n).^26^ The progression detection rate was defined as the percentage of epochs with significant responsiveness to change in the variable. The minimum wear period was defined as the smallest number of monitoring days needed to detect significant change. Slopes were calculated as dy/dt, where y=RAM or other variable, t=time. Rate of change in slopes was calculated as d^2^y/dt^2^, where y=RAM or other variable, t=time. All outcome variables were converted to z-scores for slope analysis. Correlations were assessed using Spearman’s or Pearson’s tests as appropriate. All hypothesis testing was two-tailed. A p value < 0.05 was considered significant. Mean ±SD is quoted unless otherwise stated.

## Results

### Demographics

**Figure 2.**
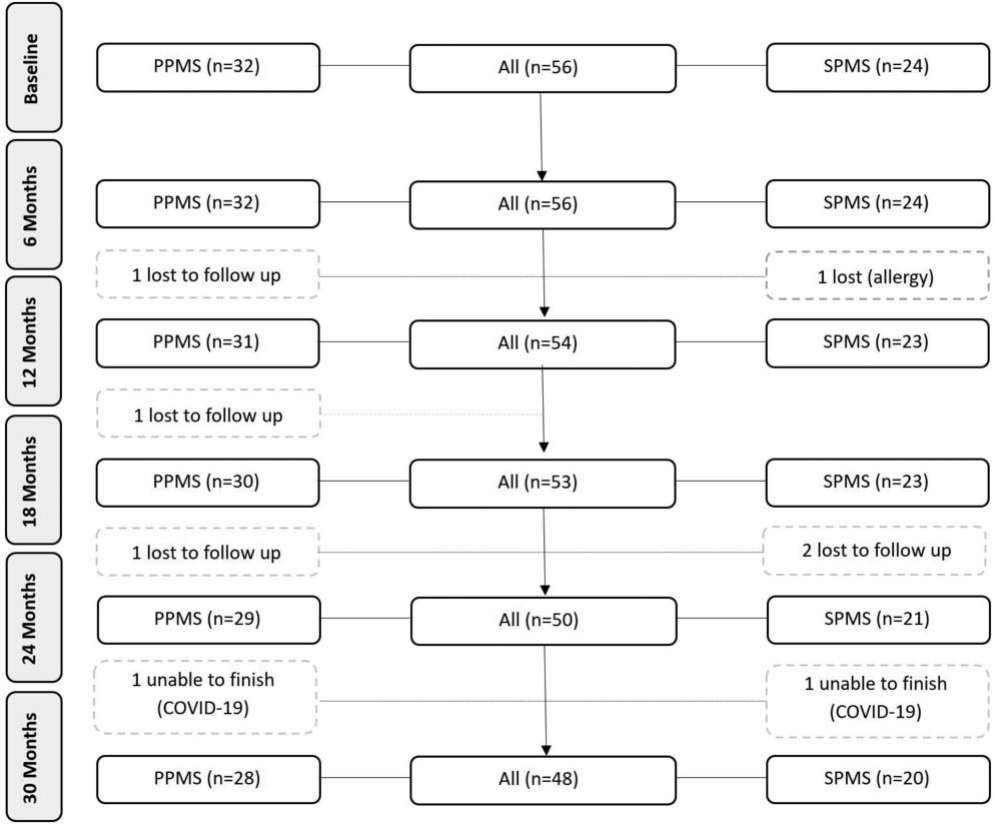
CONSORT diagram.

The CONSORT diagram is shown in Figure 2. Five participants were lost to follow-up because of negative personal life events unrelated to the study, while two could not attend their final visits due to the COVID-19 pandemic. Baseline demographic and clinical characteristics of the study population are shown in Table 1. There were significantly more males in the PPMS group (Mann Whitney U = 184, p = 0.0001) and a significantly longer disease duration in the SPMS group (Mann Whitney U = 154, p < 0.0001). SPMS study participants took significantly longer to complete a T25FW (Mann-Whitney U = 256.5, p = 0.034) and had significantly higher baseline MSIS-psych scores (Mann-Whitney U = 243, p = 0.019) compared with PPMS participants. No other baseline variables were significantly different between disease groups.

**Table 1.** Baseline demographics for the study cohort.

Demographic	All(n = 56)	PPMS(n = 32)	SPMS(n = 24)	PPMS vs. SPMS (significance, p)
Male/Female	26/30	22/10	4/20	**0.0001**
Age, years	53.6 (8.0)	53.88 (9.98)	53.13 (4.48)	0.362
Disease duration, years	12.2 (8.60)	8.82 (8.0)	16.8 (7.20)	**<0.0001**
BMI	27.9 (5.90)	28.06 (5.09)	27.74 (6.94)	0.696
EDSS	5.7 (1.30)	5.42 (1.54)	6.0 (0.84)	0.352
MSFC score	–0.017 (0.75)	–0.06 (0.71)	–0.11 (0.80)	0.540
T25FW, seconds	13.8 (11.10)	12.36 (9.54)	15.79 (12.82)	**0.034**
9-HPT, seconds	34.6 (50.50)	39.21 (66.50)	28.39 (7.80)	0.889
PASAT	43.2 (12.60)	43.75 (13.15)	42.38 (12.02)	0.551
MSIS-phys	49.0 (13.10)	47.66 (13.90)	50.75 (12.11)	0.422
MSIS-psych	18.0 (6.10)	16.31 (5.88)	20.17 (5.85)	**0.019**
Fatigue score	15.50 (6.30)	14.28 (6.63)	17.13 (5.60)	0.103
BDI	5.20 (4.70)	4.52 (4.60)	6.17 (4.79)	0.180
E_A_, joules	1401.8 (1510.6)	1460.79 (1783.87)	1323.13 (1075.34)	0.954
METs	1.30 (0.30)	1.29 (0.26)	1.28 (0.26)	0.997
PhAD, hours	1.20 (1.20)	1.19 (1.32)	1.28 (1.14)	0.663
Step count	3411.50 (2913.6)	3607.31 (3330.32)	3150.43 (2286.05)	0.85
RAM score	0.007 (0.50)	–0.01 (0.62)	0.03 (0.42)	0.889

Note: Values represent mean (SD). The differences between PPMS and SPMS baseline scores were determined using a Mann-Whitney U test.

To confirm the internal validity of the overall dataset we performed correlations between baseline characteristics. There was a moderate negative correlation between age and E_A_ (r=–0.33, p = 0.014), METs (r=–0.40, p = 0.002), PhAD (r=–0.35, p = 0.009), step count (r=–0.30, p = 0.024), and the RAM score (r=–0.32, p = 0.015) indicating that older study participants were less active. Disease duration correlated positively with baseline EDSS (r = 0.286, p = 0.034) and the T25FW (r = 0.345, p = 0.009). Body mass index (BMI) did not correlate with step count – the other RAM variables incorporated weight so their correlation with BMI was not considered. Study participants with a baseline EDSS of 6.0 or above (80% of participants) had significantly lower E_A_ scores (Mann Whitney U = 147, p = 0.046), PhAD (Mann Whitney U = 148, p = 0.048), step count (Mann Whitney U = 32, p < 0.0001), METs (Mann Whitney U = 105.5, p = 0.003), and RAM score (Mann Whitney U = 89, p = 0.001) compared to those with baseline EDSS lower than 6.0.

### Usability

RAM was well tolerated by the study participants who wore the device most of the time (median: 99.4%, interquartile range: 1.5%, mean: 99.5 ± 2.4%). Wear-times did not differ for more disabled participants (EDSS ≥ 6.0 vs EDSS < 6.0, n = 44, Mann-Whitney U = 211.5, p = 0.528). Daily wear-time did not diminish between successive six monthly assessment time points, and while it was constant during the first five days of each assessment time point (97.3 ± 1.3%), there was a slight decrease on the sixth day, to 94.9 ± 3.2% (p < 0.0001 for day, in an analysis of covariance of wear-time versus day and six monthly assessment time point, controlling for between-subject variation). One participant had an allergic reaction to the metal plates of the device after the first trial so did not participate in this sub-study while another participant developed an allergic reaction at the one-year follow-up assessment.

### RAM validation: Convergent and discriminant validity

Convergent validity of RAM was assessed using cross-sectional correlations between the RAM variables and other physical disability measures (EDSS, MSFC and MSIS-phys) at each follow-up time point (Table 2, Supplementary Table 2). RAM variables, especially step count, correlated significantly with EDSS, T25FW, 9-HPT and MSIS-phys. Discriminant validity of RAM was assessed using cross-sectional correlations between RAM variables and other scores unrelated to physical disability (PASAT, MSIS-psych, Chalder Fatigue Scale and BDI). RAM variables failed to correlate with any of these measures (Table 2 and Supplementary Table 2).

**Table 2.** Spearman’s rho correlation matrix for baseline scores, separated by those showing convergent validity (ambulatory/physical scores) and those showing discriminant validity (cognitive/psychological scores).

	Baseline correlations	E_A_	METs	PhAD	Steps	RAM score
Convergent validity
	EDSS (n = 54)	–**0.343**	–**0.411**	–**0.325**	–**0.589(<0.0001)**	–**0.419**
		**(**–**0.011)**	**(**–**0.002)**	**(**–**0.016)**		**(**–**0.002)**
	MSFC (n = 55)	**0.316**	**0.365**	**0.305**	**0.493**	**0.376**
		**(0.019)**	**(**–**0.006)**	**(**–**0.024)**	**(**–**0.0001)**	**(**–**0.005)**
	T25FW (n = 55)	–**0.331**	–**0.412**	–**0.317**	–**0.64(<0.0001)**	–**0.444**
		**(0.014)**	**(**–**0.002)**	**(**–**0.018)**		**(**–**0.001)**	
	9-HPT (n = 55)	–0.212	–0.254	–0.22	–**0.436**	–**0.321**
		(0.121)	(–0.061)	(–0.106)	**(**–**0.001)**	**(**–**0.017)**
	MSIS-phys (n = 55)	–**0.299**	–**0.287**	–**0.278**	–**0.447**	–**0.356**
		**(0.027)**	**(**–**0.033)**	**(**–**0.04)**	**(**–**0.001)**	**(**–**0.008)**
Discriminant validity	PASAT (n = 55)	0.137(0.318)	0.154	0.126	0.134	0.105
			(–0.261)	(–0.359)	(–0.329)	(–0.444)
	MSIS-psych (n = 55)	–0.142(0.303)	–0.185(–0.177)	–0.119(–0.385)	–0.234(–0.085)	–0.143(–0.297)

	Fatigue score (n = 55)	–0.163(0.235)	–0.231	–0.153	–0.203	–0.14
			(–0.09)	(–0.265)	(–0.138)	(–0.307)
	BDI (n = 54)	–0.108(0.437)	–0.052	–0.043	–0.179	–0.112
			(–0.707)	(–0.758)	(–0.195)	(–0.422)

Note: Values shown are Spearman’s r (p-value). Significant correlations are shown in bold.

### Responsiveness

**Figure 3.**
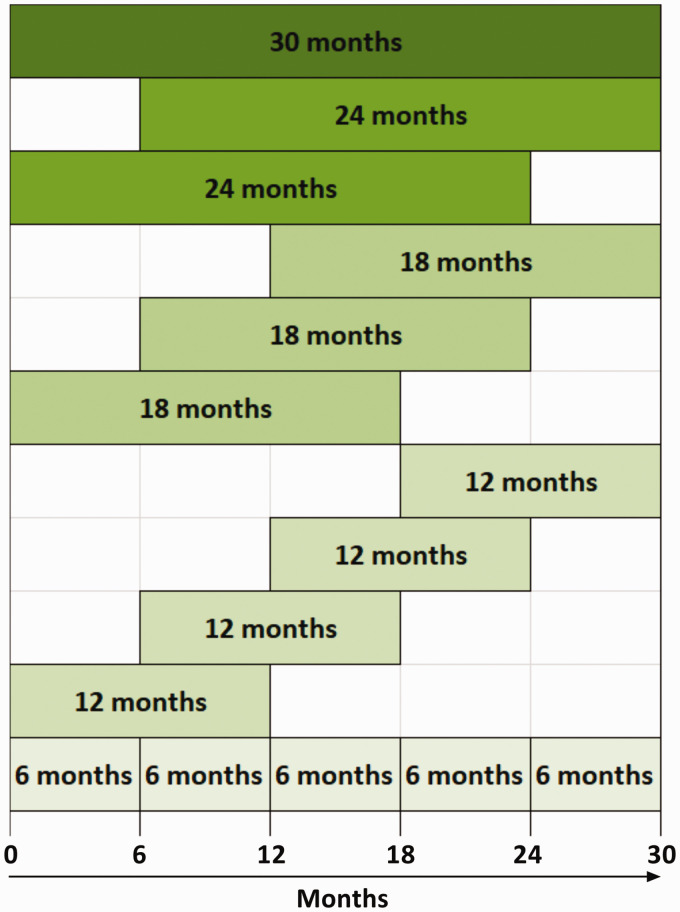
Epochs considered in the responsiveness analyses (Tables 3 and 5). All possible epochs of each duration were considered, including overlapping epochs, i.e. 15 epochs in total.

In this cohort progression was sustained, since one-sample Wilcoxon or t-tests showed that the slopes of most variables against time (dy/dt, where y = RAM variables, EDSS, MSFC or T25FW, t = time) were significantly different from zero in the direction of overall progression (Supplementary Table 3). Responsiveness of RAM, alongside the MSFC, EDSS and MSIS-phys, was assessed by the ability to detect significant (p < 0.05) change between pairs of assessments separated in time, using Wilcoxon matched pair signed rank tests. The rate of detection of progression was calculated for 6-, 12-, 18-, 24- and 30-monthly follow-up epochs, and expressed as a percentage of epochs with significant change out of all epochs (Table 3). All possible combinations of epochs were considered, including overlapping ones (Figure 3). The progression detection rate increased with longer intervals. Out of the RAM variables, step count had the highest rate of responsiveness followed by METs. RAM responsiveness was intermediate between that of the EDSS and MSFC.

**Table 3.** Percentage of epochs with significant responsiveness for EDSS, MSFC variables, MSIS-phys and RAM variables. Mean progression detection rate (%)

Follow-up period	EDSS	MSFC score	T25FW	9HPT	MSIS-phys	E_A_	METs	Step count	PhAD	RAM score
6 months (n = 5)	0	40	20	20	20	0	0	20	0	0
12 months (n = 4)	0	25	75	25	50	50	50	50	50	25
18 months (n = 3)	33.3	66.7	66.7	33.3	33.3	33.3	33.3	66.7	33.3	33.3
24 months (n = 2)	50	100	100	0	0	0	100	100	0	50
30 months (n = 1)	100	100	100	0	0	0	100	100	100	100

Note: The n-numbers in the first column refer to the number of possible epochs of a certain duration – see Figure 3. The mean progression detection rate in the bottom row represents the mean of all possible epochs of any duration i.e. 15 epochs.

Table 4 shows responsiveness for the full 30-month follow-up period. The SIMS study participants experienced a marked increase in disability with changes in all RAM variables, EDSS, MSFC (T25FW and PASAT) and fatigue scores. Participants with PPMS accumulated more disability during this study, compared to SPMS individuals. In order to investigate whether RAM responsiveness varied with disability, we repeated the analysis in participants with baseline EDSS ≥6.0 (n = 44, range: 6.0–6.5) and EDSS < 6.0 (n = 11, range: 1.5–5.5) separately. RAM was responsive in both groups (Supplementary Table 4).

**Table 4.** Responsiveness over the 30 month follow-up period for all participants.

Disability measure	Baseline median (IQR)			End median (IQR)			Significance			Effect size		
							(exact, 2-tailed)					
	All	PPMS	SPMS	All	PPMS	SPMS	All	PPMS	SPMS	All	PPMS	SPMS
EDSS	6.0 (0.5)	6.0 (1.75)	6.0(0.5)	6.5 (0.5)	6.5 (0.5)	6.5 (0.5)	**0.0005**	**0.004**	0.092	0.49	0.53	0.45
MSFC score	0.06 (1.1)	0.07 (1.09)	0.04 (1.2)	0.05 (1.27)	0.04 (1.72)	0.14 (1.13)	**0.0003**	**0.001**	0.111	0.51	0.6	0.35
T25FW, seconds	9.95 (7.61)	8.82 (5.79)	11.66 (6.48)	11.99 (10.71)	12.46 (13.66)	11.99 (7.92)	**0.0002**	**<0.0001**	0.393	0.51	0.77	0.19
9-HPT, seconds	26.92 (9.63)	26.15 (10.68)	27.44 (9.14)	25.68 (12.13)	27.46 (16.25)	24.02 (8.75)	0.067	**0.014**	0.919	0.26	0.46	0.03
PASAT	46.5 (21.75)	49.0 (21.75)	44.5 (21.75)	46.0 (24.0)	47.0 (30.25)	46.0 (15.5)	**0.035**	**0.023**	0.821	0.3	0.43	0.05
MSIS-phys	50.0 (20.5)	48.0 (20.75)	50.5 (19.0)	49.0 (17.0)	48.5 (26.25)	50.0 (15)	0.336	0.383	0.693	0.14	0.17	0.09
MSIS-psych	16.5 (9.75)	15.5 (6.75)	20.5 (11.25)	19.0 (10.0)	16.0 (10.75)	21.0 (11.0)	0.332	0.17	0.908	0.14	0.26	0.03
Fatigue score	14.0 (8.75)	13.0 (7.5)	16.5 (9.25)	16.0 (8.5)	15.0 (11.0)	18.0 (6.0)	**0.033**	**0.032**	0.499	0.3	0.4	0.15
BDI	3.0 (7.0)	3.0 (5.0)	5.0 (9.75)	5.0 (7.5)	5.0 (7.0)	4.0 (9.0)	0.854	0.244	0.372	0.03	0.22	0.2
E_A_, joules	1169.72 (1454.91)	1117.92 (1406.36)	1389.78 (1691.41)	881.31 (1321.33)	951.2 (1384.5)	837.78 (1343.38)	0.078	**0.014**	0.927	0.25	0.46	0.03
METs	1.29 (0.32)	1.25 (0.26)	1.31 (0.42)	1.22 (0.42)	1.22 (0.37)	1.23 (0.57)	**0.001**	**0.001**	0.258	0.45	0.58	0.26
PhAD, hours	0.93 (1.41)	0.9 (1.23)	0.99 (2.06)	0.73 (1.29)	0.72 (1.13)	0.73 (1.66)	**0.044**	**0.009**	0.985	0.29	0.49	0.01
Step count	2503.4 (3137.42)	2897.99 (3372.76)	2365.16 (2965.13)	1841.9 (2205.04)	1881.8 (2547.62)	1671.32 (2076)	**0.0002**	**0.001**	0.097	0.52	0.61	0.38
RAM score	0.18 (0.81)	0.18 (0.88)	0.17 (0.81)	–0.07 (0.75)	–0.03 (0.84)	–0.15 (0.69)	**0.02**	**0.003**	0.784	0.33	0.54	0.07

Note: Wilcoxon signed rank tests were used to assess if a significant change had occurred. Significant changes are in bold. Effect sizes were calculated by dividing the Wilcoxon Z by the square root of the number of observations (Z/√n).

### Change scores

**Figure 4.**
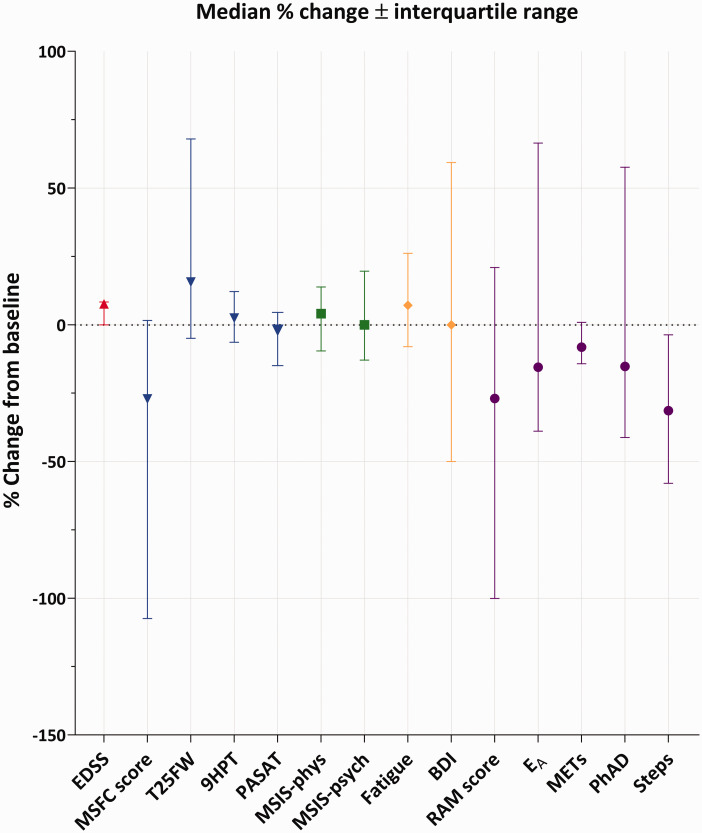
Percentage (%) change from baseline to 2.5 years, median ± interquartile range. Positive change indicates worsening for EDSS, T25FW, 9HPT, MSIS-phys, MSIS-psych, fatigue and BDI while negative change indicates worsening for the RAM variables, RAM score, MSFC score and PASAT.

Although there was good cross-sectional correlation between RAM scores and other physical measures (EDSS, T25FW, 9-HPT and MSIS-phys), there was no meaningful correlation between raw change in RAM variables and raw change in other outcome measures over the 30-month follow-up (Supplementary Table 5). This suggested that RAM measurements did not share the same progression trajectory with the other physical measures. In keeping with this explanation, there was: (1) no correlation between the slopes of the RAM variables (dy/dt, where y = RAM variable, t = time) and the other physical measures (Supplementary Table 6); (2) hardly any correlation between the rate of change of the slopes of the RAM variables (d^2^y/dt^2^, where y = RAM variable, t = time) and the other physical measures (Supplementary Table 7); (3) considerable difference in the percentage change from baseline between the different outcome measures (Figure 4).

### Brain atrophy

**Figure 5.**
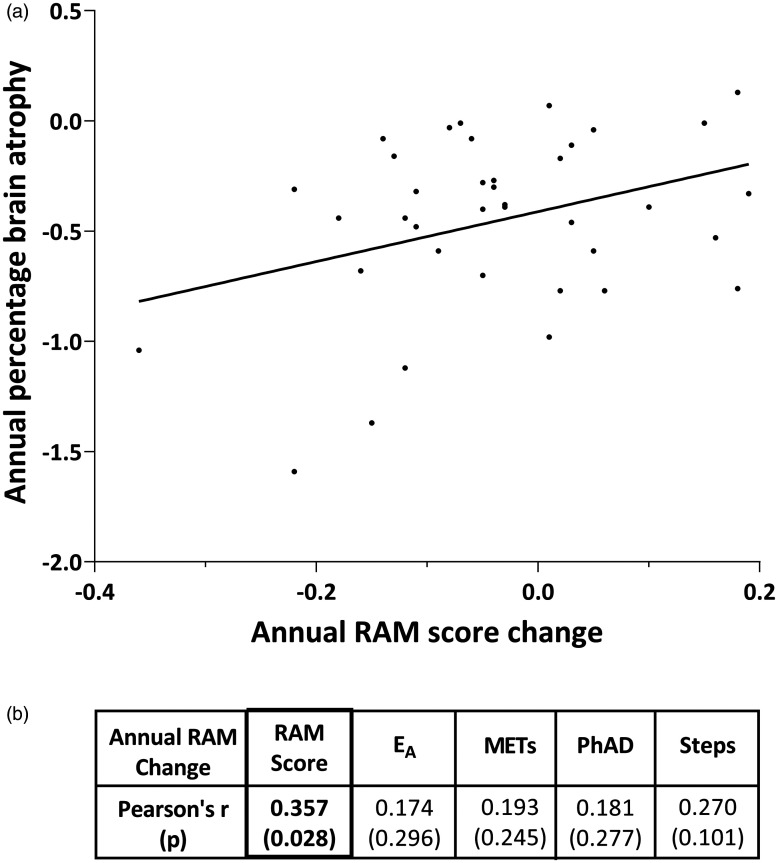
(a) Annual change in RAM score correlated with annualized percentage brain volume change while (b) individual RAM components did not correlate. E_A_ and PhAD were normalised using the cube root transformation.

In order to validate RAM against an objective structural marker of disease progression, we used annualized percentage brain volume change since brain atrophy^11^ has been used as a surrogate marker of disease progression in clinical trials.^27^ Alignment of structural brain imaging with RAM assessments within two months of each other was available for 38 study participants. The annual change in the RAM score (not individual RAM components) significantly correlated with annualized percentage brain volume change (r = 0.357, p = 0.028), (Figure 5).

### Minimum wear-period

Although participants in this study wore the device for six days, shorter periods may suffice. Since the best performing RAM score was step count, we selected this variable to assess responsiveness when the device was worn for incremental periods of time i.e. 1, 2, 3, 4, 5 and 6 days. A Kruskal-Wallis test showed no significant differences (p = 0.996) between any of the measurement periods (Supplementary Table 8). Data from the first day was as responsive as data from the entire six days (Table 5).

**Table 5.** Percentage of epochs with significant responsiveness (rows) to change in step count across different wear time durations (columns).

Follow-up period	1 day	2 days	3 days	4 days	5 days	6 days
6 months (n = 5)	0	20	40	20	20	20
12 months (n = 4)	50	75	75	50	75	50
18 months (n = 3)	100	66.7	100	66.7	100	66.7
24 months (n = 2)	100	100	100	100	100	100
30 months (n = 1)	100	100	100	100	100	100
Mean progression detection rate (%)	70.0	72.3	83.0	67.3	79.0	67.3

Note: The n-numbers in the first column refer to the number of possible epochs of a certain duration – see Figure 3. The mean progression detection rate (bottom row) is the average across all 15 epochs.

## Discussion

Remote disease monitoring is becoming increasingly desirable to reduce trips to hospitals for patient-specific, public health and environmental reasons, and the availability of patient outcome measures during remote neurology consultations would improve the quality of teleneurology.^4,28,29^ While questionnaires can be utilised for remote disease monitoring, RAM was more responsive than the three questionnaires assessed here (MSIS-29, Chalder Fatigue Scale and BDI). This study was unique since it was longitudinal and assessed multiple physical activity variables in parallel in a progressive MS population, with an assessment frequency (six monthly) matching clinical practice, and a follow-up duration sufficient to test acceptability in the long-term. We demonstrated that remote activity monitoring of individuals with MS is feasible and well tolerated including by those with higher disability, and monitoring physical activity over periods shorter than a week may suffice. We trialled posting the device to a number of participants on separate occasions after they had attended for an initial face-to-face visit to instruct them on how to use the device, and this worked well.

The RAM variables displayed very good cross-sectional correlations with all other physical measurements (EDSS, MSFC, T25FW and MSIS-phys). Despite this cross-sectional correlation between the absolute values, several strands of evidence suggest that RAM variable progression trajectories differ from those of the other physical measurements. First, RAM change scores did not correlate with changes in the EDSS, MSFC, T25FW and MSIS-phys. Second, RAM scores had different rates of change compared to the other physical measurements. Third, statistically significant change in RAM and clinically meaningful change in the other variables were not always linked (Supplementary Table 9). Fourth, two participants showed a significant decrease in step count but had no decrease in other outcome measures. In keeping with this, a recent one year study showed that a decrease in step count change may be detectable in the absence of EDSS change.^30^

RAM variables were responsive to change with step count demonstrating the highest responsiveness, and cross-sectional correlation with other outcome measures, across all time intervals assessed. This may suggest that cheaper devices, measuring steps alone, may suffice. However, the RAM score incorporating all four variables correlated with brain atrophy, while the individual RAM components did not. Hence, although change in steps may be easier to detect over shorter periods of time, the RAM score which incorporates overall physical activity and changes more slowly, is more closely related to structural brain change.

When designing this study, we planned for six days of monitoring since there was evidence that one needs this length of time for reliable measurements in healthy individuals using the device we used.^13^ Hence we were surprised to find that one day (in our case the first day was a weekday) was as responsive as six days in terms of detecting progression. Our interpretation is that people with progressive MS may have less day-to-day variability than healthy individuals, so that one day of monitoring may suffice to capture their level of disability. Although we do not have data from age and sex matched individuals to prove this, studies in MS patients using the same^14^ and other^31^ devices have shown that 2-3 days may suffice, compared to longer periods in healthy adults and children.^13,32^

This study had a number of important limitations. The use of intermittent assessment of physical activity, as opposed to continuous measurement, has the risk of bias from reactivity, i.e. a temporary increase in physical activity due to knowledge of being observed. On the other hand, intermittent assessment is more likely to be adopted in routine clinical practice. Seasonal variation may be important, since people may be more active during the summer months. Limited data was collected on the socioeconomic background or normal ambulation behaviours and environment, so activity differences related to participant lifestyles could not be controlled for. In this cohort, the mean baseline EDSS was 5.7 (range 1.5 to 6.5). While this is not unusual in progressive MS studies, it limits generalizability to early stages in MS and further study is therefore needed at lower levels of disability. Finally, brain atrophy may be influenced by factors other than MS progression (such as age and cerebrovascular disease risk factors). This study was not designed to investigate whether RAM is able to establish the relative contribution of these factors.

In conclusion, the RAM score seems a feasible and valid tool to measure progression and a potential longitudinal outcome measure in MS. Remote monitoring would reduce the need to travel for clinical appointments, reduce costs and carbon footprint, empower people with MS to participate in assessment of their condition, provide their healthcare professional with clinical outcome data during remote and/or face-to-face consultations, and facilitate longer term follow-up in research studies.

## Supplemental Material

sj-pdf-1-mso-10.1177_2055217320975185 - Supplemental material for Physical activity monitoring to assess disability progression in multiple sclerosis Click here for additional data file. Supplemental material, sj-pdf-1-mso-10.1177_2055217320975185 for Physical activity monitoring to assess disability progression in multiple sclerosis by Charlotte M Stuart, Aravinthan Varatharaj, Janine Domjan, Sheaba Philip, Ian Galea and SIMS study group in Multiple Sclerosis Journal – Experimental, Translational and Clinical

sj-pdf-2-mso-10.1177_2055217320975185 - Supplemental material for Physical activity monitoring to assess disability progression in multiple sclerosis Click here for additional data file. Supplemental material, sj-pdf-2-mso-10.1177_2055217320975185 for Physical activity monitoring to assess disability progression in multiple sclerosis by Charlotte M Stuart, Aravinthan Varatharaj, Janine Domjan, Sheaba Philip, Ian Galea and SIMS study group in Multiple Sclerosis Journal – Experimental, Translational and Clinical
