# Supplementary material for: Physical activity monitoring to assess disability progression in multiple sclerosis
Source: Mult Scler J Exp Transl Clin. 2020 Dec 7;6(4):2055217320975185. doi: 10.1177/2055217320975185 (PMC7727071; doi:10.1177/2055217320975185)
Supplement: sj-pdf-1-mso-10.1177_2055217320975185 - Supplemental material for Physical activity monitoring to assess disability progression in multiple sclerosis [file sj-pdf-1-mso-10.1177_2055217320975185.pdf]

MS Journal Appendix for MRI methodology

Hardware	
Field strength	3T
Manufacturer	Siemens
Model	Skyra
Coil type<br>(e.g. head, surface)	Head
Number of coil channels	20

Acquisition sequence		
Type<br>(e.g. FLAIR, DIR, DTI, fMRI)	MP-RAGE	
Acquisition time	5:38	
Orientation	Axial	
Alignment<br>(e.g. anterior commissure/poster commissure line)	AC-PC line	
Voxel size	1.0 x 1.0 x 1.0 mm	
TR	2200 ms	
TE	2.45 ms	
TI	900 ms	
Flip angle	8°	
NEX	1	
Field of view	250 x 250 x 176 mm	
Matrix size	250 x 250 x 176	
Parallel imaging	<u>Yes</u>	No
If used, parallel imaging method:<br>(e.g. SENSE, GRAPPA)	GRAPPA x2	
Cardiac gating	Yes	<u>No</u>
If used, cardiac gating method:<br>(e.g. PPU or ECG)		
Contrast enhancement	Yes	<u>No</u>

Acquisition sequence	
If used, provide name of contrast agent, dose and timing of scan post-contrast administration	n/a
Other parameters:	

Image analysis methods and outputs	
Lesions	
Type<br>(e.g. Gd-enhancing, T2-hyperintense, T1-hypointense)	n/a
Analysis method	
Analysis software	
Output measure<br>(e.g. count or volume [ml])	
Tissue volumes	
Type<br>(e.g. whole brain, grey matter, white matter, spinal cord)	Whole brain
Analysis method	Mean brain edge displacement
Analysis software	SIENA
Output measure<br>(e.g. absolute tissue volume in ml, tissue volume as a fraction of intracranial volume, percentage change in tissue volumes)	Percentage brain volume change
Tissue measures (e.g. MTR, DTI, T1-RT, T2-RT, T2*, T2', ^{1}H-MRS, perfusion, Na)	
Type<br>(e.g. whole brain, grey matter, white matter, spinal cord, normal-appearing grey matter or white matter)	n/a
Analysis method	
Analysis software	
Output measure	
Other MRI measures (e.g. functional MRI)	
Type<br>(e.g. whole brain, grey matter, white matter, spinal cord, normal-appearing grey matter or white matter)	n/a
Analysis method	
Analysis software	
Output measure	

Other analysis details:
