# Supplementary material for: Physical activity monitoring to assess disability progression in multiple sclerosis
Source: Mult Scler J Exp Transl Clin. 2020 Dec 7;6(4):2055217320975185. doi: 10.1177/2055217320975185 (PMC7727071; doi:10.1177/2055217320975185)
Supplement: sj-pdf-2-mso-10.1177_2055217320975185 - Supplemental material for Physical activity monitoring to assess disability progression in multiple sclerosis [file sj-pdf-2-mso-10.1177_2055217320975185.pdf]

Supplementary materials

Assessment time point	E_A	METs	PhAD	Steps
**Baseline (n=56)**	0.875 (<0.0001)	0.796 (<0.0001)	0.844 (<0.0001)	0.817 (<0.0001)
**6 months (n=55)**	0.897 (<0.0001)	0.730 (<0.0001)	0.877 (<0.0001)	0.777 (<0.0001)
**12 months (n=54)**	0.865 (<0.0001)	0.694 (<0.0001)	0.80 (<0.0001)	0.850 (<0.0001)
**18 months (n=52)**	0.871 (<0.0001)	0.691 (<0.0001)	0.837 (<0.0001)	0.762 (<0.0001)
**24 months (n=50)**	0.889 (<0.0001)	0.772 (<0.0001)	0.837 (<0.0001)	0.788 (<0.0001)
**30 months (n=48)**	0.878 (<0.0001)	0.820 (<0.0001)	0.861 (<0.0001)	0.819 (<0.0001)

Supplementary Table 1: Spearman's rho correlation coefficients (p-value) between RAM components and overall RAM scores at each assessment time point.

Baseline correlations	E_A	METs	PhAD	Steps	RAM score
**EDSS (n=54)**	**-0.343 (0.011)**	**-0.411 (0.002)**	**-0.325 (0.016)**	**-0.589 (<0.0001)**	**-0.419 (0.002)**
**MSFC (n=55)**	**0.316 (0.019)**	**0.365 (0.006)**	**0.305 (0.024)**	**0.493 (0.0001)**	**0.376 (0.005)**
**T25FW (n=55)**	**-0.331 (0.014)**	**-0.412 (0.002)**	**-0.317 (0.018)**	**-0.640 (<0.0001)**	**-0.444 (0.001)**
**9-HPT (n=55)**	-0.212 (0.121)	-0.254 (0.061)	-0.220 (0.106)	**-0.436 (0.001)**	**-0.321 (0.017)**
**MSIS-phys (n= 55)**	**-0.299 (0.027)**	**-0.287 (0.033)**	**-0.278 (0.04)**	**-0.447 (0.001)**	**-0.356 (0.008)**
**PASAT (n=55)**	0.137 (0.318)	0.154 (0.261)	0.126 (0.359)	0.134 (0.329)	0.105 (0.444)
**MSIS-psych (n=55)**	-0.142 (0.303)	-0.185 (0.177)	-0.119 (0.385)	-0.234 (0.085)	-0.143 (0.297)
**Fatigue score (n=55)**	-0.163 (0.235)	-0.231 (0.09)	-0.153 (0.265)	-0.203 (0.138)	-0.14 (0.307)
**BDI (n=54)**	-0.108 (0.437)	-0.052 (0.707)	-0.043 (0.758)	-0.179 (0.195)	-0.112 (0.422)

6 month correlations	E_A	METs	PhAD	Steps	RAM score
**EDSS (n=39)**	-0.140 (0.396)	-0.270 (0.097)	-0.125 (0.45)	**-0.355 (0.027)**	-0.236 (0.147)
**MSFC (n=55)**	0.265 (0.050)	0.225 (0.099)	0.249 (0.066)	**0.316 (0.019)**	**0.342 (0.011)**
**T25FW (n=55)**	**-0.30 (0.026)**	**-0.282 (0.037)**	**-0.282 (0.037)**	**-0.497 (0.0001)**	**-0.421 (0.001)**
**9-HPT (n=55)**	-0.147 (0.283)	-0.181 (0.187)	-0.161 (0.240)	**-0.333 (0.013)**	**-0.284 (0.035)**
**MSIS-phys (n=54)**	-0.260 (0.057)	-0.288 (0.034)	-0.246 (0.073)	**-0.346 (0.010)**	**-0.323 (0.017)**
**PASAT (n=55)**	0.143 (0.298)	0.005 (0.974)	0.099 (0.472)	-0.039 (0.780)	0.102 (0.457)
**MSIS-psych (n=54)**	-0.071 (0.609)	-0.129 (0.352)	-0.075 (0.592)	-0.155 (0.263)	-0.074 (0.593)
**Fatigue score (n=55)**	-0.184 (0.18)	-0.239 (0.079)	-0.228 (0.095)	-0.215 (0.114)	-0.181 (0.187)
**BDI (n=55)**	-0.013 (0.923)	-0.098 (0.475)	-0.003 (0.985)	-0.153 (0.264)	-0.041 (0.768)

12 month correlations	E_A	METs	PhAD	Steps	RAM score
**EDSS (n=44)**	**-0.381 (0.011)**	**-0.370 (0.013)**	**-0.387 (0.009)**	**-0.514 (0.003)**	**-0.433 (0.003)**
**MSFC (n=54)**	**0.330 (0.015)**	**0.276 (0.043)**	**0.314 (0.021)**	**0.504 (0.0001)**	**0.395 (0.003)**
**T25FW (n=54)**	**-0.432 (0.001)**	**-0.399 (0.003)**	**-0.405 (0.002)**	**-0.635 (<0.0001)**	**-0.512 (<0.0001)**
**9-HPT (n=53)**	-0.235 (0.09)	-0.184 (0.188)	-0.230 (0.097)	**-0.492 (0.0002)**	**-0.399 (0.003)**
**MSIS-phys (n=54)**	-0.235 (0.088)	-0.233 (0.089)	-0.206 (0.135)	**-0.456 (0.001)**	**-0.341 (0.012)**
**PASAT (n=54)**	0.116 (0.404)	0.08 (0.566)	0.122 (0.378)	0.032 (0.817)	0.027 (0.844)
**MSIS-psych (n=54)**	-0.002 (0.986)	-0.021 (0.88)	0.014 (0.922)	-0.175 (0.205)	-0.096 (0.491)
**Fatigue score (n=54)**	-0.111 (0.426)	-0.11 (0.429)	-0.105 (0.45)	-0.159 (0.25)	-0.136 (0.328)
**BDI (n=54)**	-0.126 (0.363)	-0.099 (0.477)	-0.104 (0.452)	**-0.273 (0.046)**	-0.174 (0.208)

18 month correlations	E_A	METs	PhAD	Steps	RAM score
**EDSS (n=28)**	-0.284 (0.136)	-0.244 (0.203)	-0.253 (0.185)	**-0.585 (0.001)**	**-0.498 (0.006)**
**MSFC (n=49)**	0.241 (0.085)	0.199 (0.158)	0.246 (0.079)	**0.495 (0.0002)**	**0.476 (0.0004)**
**T25FW (n=49)**	**-0.328 (0.018)**	**-0.304 (0.028)**	**-0.329 (0.017)**	**-0.543 (<0.0001)**	**-0.490 (0.0002)**
**9-HPT (n=49)**	**-0.347 (0.013)**	**-0.357 (0.011)**	**-0.371 (0.008)**	**-0.510 (0.0002)**	**-0.534 (<0.0001)**
**MSIS-phys (n=48)**	**-0.334 (0.019)**	-0.273 (0.057)	**-0.353 (0.013)**	**-0.406 (0.004)**	**-0.462 (0.001)**
**PASAT (n=49)**	0.085 (0.559)	-0.064 (0.661)	0.052 (0.72)	0.043 (0.766)	0.176 (0.221)
**MSIS-psych (n=48)**	0.009 (0.951)	0.047 (0.746)	-0.007 (0.964)	-0.082 (0.574)	-0.067 (0.647)
**Fatigue score (n=48)**	0.024 (0.868)	0.052 (0.722)	0.003 (0.984)	0.014 (0.926)	0.023 (0.876)
**BDI (n=48)**	-0.020 (0.892)	-0.037 (0.8)	-0.026 (0.86)	-0.163 (0.263)	-0.110 (0.453)

24 month correlations	E_A	METs	PhAD	Steps	RAM score
**EDSS (n=42)**	-0.30 (0.053)	**-0.320 (0.039)**	-0.275 (0.077)	**-0.679 (<0.0001)**	**-0.520 (0.0004)**
**MSFC (n=50)**	**0.304 (0.032)**	**0.352 (0.012)**	**0.301 (0.033)**	**0.629 (<0.0001)**	**0.507 (0.0002)**
**T25FW (n=50)**	**-0.347 (0.014)**	**-0.374 (0.008)**	**-0.310 (0.029)**	**-0.668 (<0.0001)**	**-0.514 (0.0001)**
**9-HPT (n=50)**	-0.205 (0.153)	**-0.332 (0.018)**	-0.216 (0.131)	**-0.550 (<0.0001)**	**-0.403 (0.004)**
**MSIS-phys (n=50)**	**-0.353 (0.012)**	**-0.383 (0.006)**	**-0.373 (0.008)**	**-0.433 (0.002)**	**-0.440 (0.001)**
**PASAT (n=50)**	0.162 (0.26)	0.098 (0.498)	0.144 (0.317)	0.165 (0.252)	0.240 (0.093)
**MSIS-psych (n=50)**	-0.211 (0.141)	-0.217 (0.130)	-0.206 (0.152)	-0.148 (0.306)	-0.168 (0.244)
**Fatigue score (n=50)**	-0.059 (0.684)	-0.149 (0.303)	-0.063 (0.665)	-0.156 (0.28)	-0.085 (0.557)
**BDI (n=49)**	-0.156 (0.284)	-0.196 (0.178)	-0.175 (0.229)	-0.235 (0.104)	-0.186 (0.20)

30 month correlations	E_A	METs	PhAD	Steps	RAM score
**EDSS (n=43)**	**-0.347 (0.019)**	**-0.375 (0.011)**	**-0.363 (0.014)**	**-0.665 (<0.0001)**	**-0.609 (<0.0001)**
**MSFC (n=46)**	**0.376 (0.008)**	**0.421 (0.003)**	**0.399 (0.005)**	**0.599 (<0.0001)**	**0.588 (<0.0001)**
**T25FW (n=46)**	**-0.484 (0.0004)**	**-0.510 (0.0002)**	**-0.487 (0.0004)**	**-0.708 (<0.0001)**	**-0.705 (<0.0001)**
**9-HPT (n=46)**	**-0.390 (0.006)**	**-0.373 (0.009)**	**-0.401 (0.005)**	**-0.443 (0.002)**	**-0.496 (0.0003)**
**MSIS-phys (n=46)**	**-0.295 (0.042)**	**-0.346 (0.016)**	**-0.304 (0.036)**	**-0.378 (0.008)**	**-0.437 (0.002)**
**PASAT (n=46)**	0.167 (0.257)	0.135 (0.361)	0.170 (0.247)	0.154 (0.295)	0.250 (0.086)
**MSIS-psych (n=46)**	-0.138 (0.35)	-0.222 (0.13)	-0.127 (0.39)	-0.103 (0.487)	-0.166 (0.260)
**Fatigue score (n=46)**	-0.207 (0.157)	-0.239 (0.102)	-0.222 (0.130)	-0.122 (0.407)	-0.216 (0.140)
**BDI (n =46)**	-0.124 (0.401)	-0.139 (0.345)	-0.108 (0.463)	-0.088 (0.550)	-0.159 (0.280)

Supplementary Table 2: Spearman's rho correlation coefficients (p-value) between scores at all assessment time points. Significant correlations are highlighted in bold.

Disability measure	N	Mean (or Median*) slope	SD (or IQR*)	Significance (2-tailed)
**EDSS***	54	-0.08*	0.18*	**0.007**
**MSFC score***	55	-0.05*	0.19*	**0.002**
**T25FW, seconds***	50	-0.07*	0.21*	**<0.0001**
**9-HPT, seconds***	55	-0.05*	0.23*	0.074
**PASAT***	55	-0.01*	0.21*	0.257
**MSIS-phys**	55	-0.04	0.25	0.254
**MSIS-psych***	55	0.0*	0.33*	0.527
**Fatigue score**	55	-0.05	0.36	0.267
**BDI***	55	0.01*	0.39*	0.912
**E_A, joules**	54	-0.09	0.27	**0.023**
**METs**	54	0.11	0.29	**0.006**
**PhAD, hours**	54	-0.09	0.23	**0.008**
**Step count**	54	-0.15	0.24	**<0.0001**
**RAM score***	54	-0.05*	0.15*	**0.026**

*Supplementary Table 3: To assess if overall progression was significantly different from zero, one sample t-tests (for parametric data) or one sample Wilcoxon tests (for non-parametric data, denoted by *) were employed. Mean (*or median) slopes of variables against time (dy/dt, where y=variables of disability measures, t=time) and standard deviation (*or interquartile range, IQR) are shown with significant changes (p values) highlighted in bold. All outcome variables were converted to z-scores for this analysis, therefore a negative value represents worsening of scores for all outcome variables.*

Disability measure	Baseline median (IQR)		End median (IQR)		Significance (exact, 2-tailed)		Effect size	
	EDSS <6.0	EDSS ≥6.0	EDSS <6.0	EDSS ≥6.0	EDSS <6.0	EDSS ≥6.0	EDSS <6.0	EDSS ≥6.0
**EDSS**	3.5 (2.5)	6.0 (0.5)	4.0 (4.0)	6.5 (0.5)	0.15	**0.0004**	0.48	0.59
**MSFC score**	0.73 (0.71)	-0.11 (1.06)	0.53 (0.78)	-0.23 (1.55)	0.495	**0.0004**	0.24	0.56
**T25FW, seconds**	5.95 (2.24)	11.62 (9.27)	5.94 (2.66)	14.75 (14.37)	0.10	**0.0003**	0.53	0.56
**9-HPT, seconds**	22.21 (7.58)	27.58 (10.34)	23.98 (8.79)	26.56 (14.21)	0.32	0.14	0.34	0.24
**PASAT**	50.0 (8.0)	44.5 (23.0)	51.5 (13.0)	45.5 (30.25)	0.90	**0.008**	0.05	0.42
**MSIS-phys**	36.0 (22)	53.0 (13.75)	34.5 (19.25)	53.5 (19.5)	0.54	0.48	0.21	0.12
**MSIS-psych**	14.0 (6.0)	18.0 (10.5)	15.0 (10.25)	20.0 (10.75)	0.63	0.50	0.23	0.11
**Fatigue score**	11.0 (9.0)	14.0 (9.0)	13.5 (6.5)	16.5 (8.0)	**0.02**	0.24	0.75	0.19
**BDI**	2.0 (2.0)	4.0 (7.0)	2.5 (6.0)	5.0 (8.0)	0.59	0.89	0.2	0.02
**E_A, joules**	1434.97 (545.53)	1018.41 (1627.61)	1153.77 (1292.15)	769.27 (1383.13)	0.06	0.31	0.6	0.17
**METs**	1.44 (0.13)	1.21 (0.36)	1.32 (0.17)	1.18 (0.42)	**0.03**	**0.01**	0.69	0.42
**PhAD, hours**	1.36 (1.11)	0.84 (1.57)	1.05 (0.91)	0.65 (1.27)	**0.05**	0.23	0.63	0.2
**Step count**	6636.75 (2926.67)	2190.23 (1767.83)	4273.75 (3704.14)	1480.58 (1790.39)	**0.009**	**0.003**	0.79	0.47
**RAM score**	0.42 (0.27)	-0.07 (0.79)	0.22 (0.48)	-0.25 (0.76)	0.08	0.06	0.56	0.31

Supplementary Table 4: Responsiveness over the 30 month follow-up period: sensitivity analysis by EDSS <6.0 and EDSS ≥6.0. Wilcoxon signed rank tests were used to assess if a significant change had occurred. Significant changes are in bold. Effect sizes were calculated by dividing the Wilcoxon Z by the square root of the number of observations ($Z/\sqrt{n}$).

Change correlations	E_A	METs	PhAD	Steps	RAM score
**EDSS (n=47)**	0.065 (0.663)	-0.196 (0.187)	0.005 (0.975)	-0.128 (0.390)	-0.032 (0.833)
**MSFC (n=50)**	-0.191 (0.184)	-0.09 (0.532)	-0.17 (0.238)	-0.091 (0.531)	-0.11 (0.449)
**T25FW (n=50)**	0.155 (0.282)	0.024 (0.867)	0.137 (0.343)	0.076 (0.6)	0.007 (0.964)
**9-HPT (n=50)**	0.178 (0.216)	0.088 (0.543)	0.203 (0.157)	0.093 (0.519)	0.124 (0.391)
**MSIS-phys (n=50)**	0.197 (0.171)	0.168 (0.243)	0.182 (0.207)	0.014 (0.922)	0.129 (0.374)
**PASAT (n=50)**	0.140 (0.333)	0.140 (0.332)	0.165 (0.253)	0.178 (0.215)	0.176 (0.221)
**MSIS-psych (n=50)**	0.226 (0.114)	0.154 (0.284)	0.186 (0.195)	0.272 (0.056)	**0.306 (0.031)**
**Fatigue Score (n=50)**	0.046 (0.749)	0.098 (0.498)	0.057 (0.693)	0.013 (0.931)	0.108 (0.456)
**BDI (n=49)**	**0.295 (0.039)**	0.075 (0.608)	0.250 (0.083)	0.246 (0.088)	**0.356 (0.012)**

Supplementary Table 5: Spearman's rho correlation coefficients (p-value) between change scores. Significant correlations are highlighted in bold.

Slope correlations	E_A	METs	PhAD	Steps	RAM score
**EDSS (n=53)**	0.111 (0.427)	0.009 (0.949)	0.118 (0.399)	0.163 (0.244)	0.150 (0.282)
**MSFC (n=54)**	-0.023 (0.87)	0.103 (0.46)	-0.041 (0.771)	-0.049 (0.727)	-0.094 (0.498)
**T25FW (n=54)**	0.062 (0.656)	0.064 (0.648)	0.057 (0.683)	0.221 (0.109)	0.110 (0.428)
**9-HPT (n=54)**	0.174 (0.209)	-0.080 (0.567)	0.125 (0.37)	0.125 (0.368)	0.054 (0.701)
**MSIS-phys (n=54)**	-0.007 (0.962)	0.079 (0.572)	-0.011 (0.936)	0.026 (0.853)	-0.020 (0.885)
**PASAT (n=54)**	-0.130 (0.351)	0.092 (0.507)	-0.145 (0.296)	0.125 (0.368)	-0.024 (0.861)
**MSIS-psych (n=54)**	0.168 (0.23)	-0.034 (0.807)	0.179 (0.2)	-0.002 (0.989)	0.023 (0.871)
**Fatigue Score (n=54)**	0.101 (0.466)	-0.029 (0.836)	0.102 (0.461)	-0.053 (0.702)	-0.013 (0.925)
**BDI (n=54)**	0.002 (0.988)	-0.082 (0.554)	0.011 (0.935)	-0.117 (0.399)	-0.166 (0.231)

Supplementary Table 6: Spearman's rho correlation coefficients (p-value) between the slopes of the outcome variables over the 30-month follow-up period. All outcome variables were converted to z-scores for this analysis.

Rate of change of slopes correlations	E_A	METs	PhAD	Steps	RAM score
**EDSS (n=43)**	0.044 (0.779)	**-0.399 (0.008)**	0.043 (0.783)	0.150 (0.337)	-0.085 (0.589)
**MSFC (n=50)**	-0.128 (0.367)	-0.106 (0.456)	-0.136 (0.337)	-0.109 (0.441)	-0.17 (0.228)
**T25FW (n=50)**	0.009 (0.951)	-0.031 (0.83)	-0.007 (0.959)	0.253 (0.071)	-0.02 (0.889)
**9-HPT (n=50)**	-0.209 (0.137)	-0.129 (0.361)	-0.207 (0.141)	-0.133 (0.346)	-0.271 (0.052)
**MSIS-phys (n=50)**	**0.279 (0.047)**	-0.189 (0.184)	0.273 (0.052)	**0.343 (0.014)**	0.245 (0.084)
**PASAT (n=50)**	-0.128 (0.365)	0.136 (0.335)	-0.129 (0.361)	0.01 (0.946)	-0.089 (0.529)
**MSIS-psych (n=50)**	0.244 (0.082)	**-0.332 (0.016)**	0.255 (0.068)	**0.274 (0.05)**	0.206 (0.143)
**Fatigue Score (n=50)**	0.102 (0.472)	0.046 (0.747)	0.066 (0.64)	-0.06 (0.674)	0.141 (0.319)
**BDI (n=50)**	0.183 (0.193)	0.003 (0.984)	0.195 (0.165)	0.222 (0.114)	0.211 (0.132)

Supplementary Table 7: Spearman's rho correlation coefficients (p-value) between the rate of change of slopes of the outcome variables. Slopes were calculated for every six monthly epoch except for EDSS which was calculated using two 18 month epochs. Significant correlations are highlighted in bold. All outcome variables were converted to z-scores for this analysis.

Averaged over:	N	Mean	SD	Mean rank	Mean rank difference	Z	Significance, (approximate, 2-tailed)
**One Day**	326	2890.1	2674.0	966.1	11.2	0.253	>0.9999
**Two Days**	326	3005.9	2731.3	988.8	-11.5	0.260	>0.9999
**Three Days**	326	3017.3	2771.8	987.6	-10.3	0.234	>0.9999
**Four Days**	326	2957.5	2745.8	977.4	-0.1	0.002	>0.9999
**Five Days**	326	2925.5	2683.8	973.7	3.6	0.082	>0.9999
**Six Days**	326	2912.9	2604.8	977.3	-	-	-

Supplementary Table 8: A Kruskal-Wallis test was performed to assess whether there was a difference in the mean step count between RAM device wear duration of six days versus shorter periods (p=0.996).

Time points (months)	6	12	18	24	30
E_A	-	+	+	-	-
METs	-	+	+	+	+
PhAD	-	+	+	-	+
Steps	-	-	+	+	+
RAM score	-	-	+	+	+
EDSS[1]	-	+	+	-	+
MSFC[2]	-	-	-	-	+
T25FW[2]	-	-	-	-	+
9HPT[2]	-	-	-	-	-
PASAT[2]	-	-	-	-	-
MSIS-phys[3]	-	-	-	-	-
MSIS-psych[4]	-	-	-	-	-

Supplementary Table 9: RAM variable responsiveness was assessed using Wilcoxon signed rank tests, at each time point corresponding to a clinic visit (i.e. at 6, 12, 18, 24 and 30 months), compared to baseline. If the RAM variable was significantly responsive at that time point (p<0.05), a "+" is shown, otherwise a "-" is shown. For the other variables the median value at each time point was compared to baseline medians. If the variable had exhibited a minimum clinically important difference (MID) [1-4], a "+" is shown, otherwise a "-" is shown.

References

1. Kurtzke JF. Rating neurologic impairment in multiple sclerosis: an expanded disability status scale (EDSS). *Neurology* 1983;33(11):1444-52. doi: 10.1212/wnl.33.11.1444
2. Rudick RA, Polman CH, Cohen JA, et al. Assessing disability progression with the Multiple Sclerosis Functional Composite. *Multiple sclerosis (Houndmills, Basingstoke, England)* 2009;15(8):984-97. doi: 10.1177/1352458509106212 [published Online First: 2009/08/12]
3. Costelloe L, O'Rourke K, Kearney H, et al. The patient knows best: significant change in the physical component of the Multiple Sclerosis Impact Scale (MSIS-29 physical). *Journal of Neurology, Neurosurgery & Psychiatry* 2007;78(8):841-44. doi: 10.1136/jnnp.2006.105759
4. McGuigan C, Hutchinson M. The multiple sclerosis impact scale (MSIS-29) is a reliable and sensitive measure. *J Neurol Neurosurg Psychiatry* 2004;75(2):266-69.
